# Commencement of the Atlanta School of Medicine

**Published:** 1908-06

**Authors:** 


### COMMENCEMENT OF THE ATLANTA SCHOOL OF MEDICINE.

The Third Annual Commencement of the Atlanta School of Medicine was held April 22nd, in the Grand Opera House.

The conferring of degrees was by Ex-Gov. W. J. Northen, president of the board of trustees, and the annual address by Dr. John G. Olmstead. The report of conditions at the college, rendered by Dr George H. Noble, showed an enrollment of 274 students—an increase of 18 per cent. over that of last year. The graduates numbered 49.

Seven seniors were the recipients of certificates of distinction. They are as follows: W. C. Hays, W. C. Tipton, C. D. Bannister, O. O. Fanning, R. A. Verier, S. E. Clinard, I. Willis.

Those receiving the degree Doctor of Medicine, were:

From Georgia: W. D. Aderhold, C. D. Bannister, J. H. Barkwell, B. S. Bomar, J. C. Bramblett, J. B. Brown, A. B. Burns, O. B. Bush, H. O. Byrd (president of class), S. E. Clinard, Y. R. Coleman, W. J. Creel, W. L. Ethridge, J. K. Hall, W. C. Hays, F. B. Hill, W. H. Houston, W. D. Howard, N. B. Hutchison, W. A. N. Jones, A. Lazenby, W. H. Lewis, J. E. Morrison, G. E. Neal, C. E. New, G. S. Sellman, W. C. Tipton, T. G. Turk, H. G. Wallace, H. H. H. Ward, C. W. White, L. W. Wiggins, R. P.Orton, H. C. Stovall, T. L. Vineyard. From Florida: D. H. Adams, O. O. Fannings; A. S. Applewhite, Mississippi; M. Medlin, Mississippi; G. L. Webb, Mississippi; E. L. Patterson, South Carolina; R. A. Verdier, South Carolina; O. C. Grunitz, Tennessee; J. A. Saliba, Turkey.

